# A Canadian multi-province study of COVID-19 vaccine coverage along area-level social determinants in 2021

**DOI:** 10.1016/j.puhip.2025.100594

**Published:** 2025-02-20

**Authors:** Reed F. Beall, Jorge Luis Flores Anato, Adam G. D'Souza, Héctor Alexander Velásquez García, Huiting Ma, Fengjuan Yang, Stefan D. Baral, Jason Cabaj, Elizabeth Cooper, Aidan Hollis, Naveed Zafar Janjua, Alan Katz, Jenine Leal, Mathieu Maheu-Giroux, Elissa Rennert May, Kamil Malikov, Sharmistha Mishra, Gary Moloney, Tyler Williamson

**Affiliations:** a Department of Community Health Sciences, Cumming School of Medicine, University of Calgary, Calgary, Alberta, Canada; b O'Brien Institute for Public Health, University of Calgary, Canada; c Department of Epidemiology and Biostatistics, School of Population and Global Health, McGill University, Montréal, Quebec, Canada; d Centre for Health Informatics, Cumming School of Medicine, University of Calgary, Calgary, Alberta, Canada; e Provincial Research Data Services, Alberta Health Services, Alberta, Canada; f University of British Columbia Centre for Disease Control, Vancouver, Canada; g Data and Analytic Services, British Columbia Centre for Disease Control, Vancouver, Canada; h MAP Centre for Urban Health Solutions, St. Michael's Hospital, Unity Health Toronto, Toronto, Ontario, Canada; i Department of Epidemiology, Johns Hopkins School of Public Health, Baltimore, MD, USA; j Population and Public Health, Alberta Health Services, Alberta, Canada; k Faculty of Kinesiology and Health Studies, University of Regina, Saskatchewan, Canada; l Ontario Ministry of Health, Toronto, Canada; m Department of Economics, University of Calgary, Alberta, Canada; n School of Population and Public Health, University of British Columbia, Vancouver, Canada; o Manitoba Centre for Health Policy, Community Health Sciences and Family Medicine, Max Rady College of Medicine, University of Manitoba, Winnipeg, Manitoba, Canada; p Department of Microbiology, Immunology, and Infectious Diseases, Cumming School of Medicine, University of Calgary, Canada; q Infection Prevention and Control, Alberta Health Services, Canada; r Division of Infectious Diseases, Department of Medicine, University of Toronto, Toronto, ON, Canada; s St Michael's Hospital, Unity Health Toronto, Toronto, ON, Canada

**Keywords:** Canada, Cross-sectional studies, COVID-19 vaccines∗, COVID-19∗, Epidemiology, Prevention & control, Humans, Observational, Vaccination

## 1 Introduction

The COVID-19 pandemic exemplified the relationship between social inequities and the distribution of infectious diseases [1,2]. Health inequities represent unfair and avoidable differences in health outcomes closely linked to disparities in the social, economic, and environmental conditions [3]. Canadian federal and provincial health authorities invested significant resources into executing predominantly age-centered vaccine rollout strategies with only a few informed by health inequities frameworks [4].

Once COVID-19 vaccines became widely available in the spring of 2021, provincial governments and health authorities began to recognize that socially disadvantaged neighbourhoods were often high-transmission areas with less vaccine availability. Therefore, some public health authorities began prioritizing vaccination access in these areas to improve coverage [[5], [6], [7], [8]]. Meanwhile, other factors simultaneously aided access to COVID-19 vaccines in privileged neighbourhoods. For example, pharmacies, which have a more significant presence in higher income neighbourhoods, joined as major players in vaccine rollout strategies in March 2021 [9,10]. Social media groups like “vaccine hunters” were formed, which may have facilitated faster uptake among digitally literate early adopters over their less advantaged peers [11].

Given these contravening factors reinforcing or contradicting social inequities, as well as the likely variable impact of provincial policies to speed vaccination uptake (e.g., vaccine passports, workplace mandates) [4], exploring how coverage evolved and differed between social groups could be useful for informing future vaccination campaigns. Similar within-country or within-region analyses have been conducted internationally, including in non-high-income countries, highlighting shared challenges in addressing vaccination disparities while emphasizing the importance of context-specific solutions [12,13]. While prior studies have investigated this subject in a single jurisdiction [14,15] or have focused on specific vaccine policies [16], none have broadly compared neighbourhood-level disparities in first-dose and second-dose COVID-19 vaccination coverage in 2021 across Canadian provinces. Therefore, our objective was to explore the rise of COVID-19 vaccination coverage across the four largest Canadian provinces of Alberta, British Columbia, Ontario, and Quebec, focusing on a range of area-level markers for social and economic advantage.

## 2 Methods

### 2.1 Design

We descriptively examine the cumulative changes in first- and second-dose vaccine coverage and vaccination rates throughout 2021 in groups of neighbourhoods ranked by their level of social advantage according to six area-level social determinants of health.

### 2.2 Data

To compile a multi-province dataset, we used an approach previously used to examine heterogeneity in COVID-19 risk across provinces [1,2]. Provincial policies restrict cross-provincial sharing of individual-level and full postal code or census dissemination area (DA) level data, which typically represent approximately 400–700 people. However, all provinces allowed sharing of data when neighbourhoods (i.e., Census Dissemination Areas [DAs]) were placed into ranked bins (e.g., quintiles), so that the geographic regions were large enough to protect residents’ privacy while still creating informative groupings. Therefore, each province designated an analyst to access their vaccine registries and sum weekly first- and second-dose vaccine administrations by postal code in 2021. The provincial immunization data sources were the Immunization Administration Record Inventory (Alberta), ImmunizeBC (British Columbia), COVaxON (Ontario), and the Registre de vaccination du Québec (Quebec). Data from Quebec were available only for residents aged 12+ years. All other provinces reported all vaccinations without age restrictions.

After aligning postal codes and DAs using Statistics Canada's 2016 postal code conversation (PCCF+) file [17], the analysts used a common codebase to rank areas into quintiles based on social and structural determinants (described below). We focused on Canada's four most-populated provinces, encompassing 86.5 % of the total Canadian population.

### 2.3 Ethics approvals

Ethics approvals were obtained from the University of British Columbia Research Ethics Board (H20-02097), the University of Calgary Health Research Ethics Board (REB20-0688, REB19-1369_MOD1) and the Health Information Privacy Committee of Alberta Health (No. 2020/2021-32), the University of Toronto Health Sciences Research Ethics Board (39253) and the Institutional Review Board of McGill University in Quebec (A06-M52-20B).

### 2.4 Covariates: social and structural determinants of health

Using Statistics Canada's Census data (2016) available at the time of database construction [18], each analyst ranked areas on factors previously observed to be associated with elevated COVID risk [2]: (i) average household income (after-tax) and the fractions of (ii) adults with high school diplomas, (iii) recent immigrants, (iv) visible minorities, (v) suitable housing, and (vi) those with occupations amenable to remote working. For each variable, the analysts sorted (highest to lowest) neighbourhoods by within-province ranks, and then assigned a quintile (1 = least-advantaged, 5 = most-advantaged). Each indicator of social or economic advantage was ranked and analyzed separately. For example, the top 20 percent of the provincial population residing in neighbourhoods with the highest proportions of high school graduates in the province.

### 2.5 Outcomes

i) Weekly first- and second-dose vaccination rates

To calculate vaccination rates, we used the total census population as the denominator. We adjusted the denominator for each week by subtracting the cumulative sum of first- and second-dose vaccinations received by those living in that same region in prior weeks. The weekly incidence rate was then computed as the number of first- or second-doses received (as the numerator) divided by the remaining total population that were unvaccinated multiplied by 10,000. This indicator provides perspective on the degree of absolute difference in the vaccination rate across quintiles and time.

ii) Cumulative vaccination coverage, first- and second-dose vaccinations

To calculate weekly cumulative vaccination coverage, we summed all vaccines received per neighbourhood and week plus all others previously received. This cumulative total was then divided by the total neighbourhood population. This indicator provides perspective on the absolute cumulative differences in the rate of vaccination across quintiles and time.

iii) Gini coefficient scores for the number of vaccinations across quintiles and time

To track changes in the relative proportions of vaccinations over time, we calculated Gini coefficients based on the number of new vaccinations per 10,000 persons per quintile. Traditionally, Gini coefficients range between 0 and 1, depending on the deviation from perfect cumulative proportionality (e.g., the lowest ranking 20 percent of the population has 20 percent of vaccination administrations, the lowest 40 percent has 40 percent of vaccination administrations, and so on). A perfectly proportionate distribution has a Gini coefficient of 0. Values closer to 1 suggest vaccines were disproportionately allocated to higher ranking populations. Gini coefficients below 0.20 are considered a low degree of inequality, 0.25–0.35 is moderate, 0.35–0.50 is high, and over 0.50 is extreme [19]. We allowed for negative Gini coefficient values to represent scenarios in which disadvantaged areas had disproportionately more vaccinations than advantaged populations. Similar adjustments to the traditional Gini coefficient measure have been described elsewhere as a Concentration Index; however, this is a less familiar term to many audiences [20].

### 2.6 Analysis and sensitivity analysis

We plotted weekly provincial incidence rates, diffusion curves, and Gini coefficients for first- and second-doses in 2021, using R statistical software (version 4.2.1) and ggplot2 (version 3.3.6). While data sharing restrictions did not allow for stratification by urban versus rural in all provinces, the study team focused on Alberta to test the extent to which such a distinction would change observed patterns, given that rurality may have been an important driver of unique patterns in some provinces. To work within data sharing restrictions, we reran all analyses for Calgary and Edmonton's metropolitan areas only (based on the census regions) since those populations were large enough to avoid raising any privacy concerns. We then compared Alberta's main analysis to its urban one to evaluate differences.

## 3 Results

Our final dataset included 46,872 neighbourhoods (DAs), housing 29,600,926 individuals–approximately 84.2 % of the overall Canadian population based on available census data (Table 1). The neighbourhood quintiles had a pooled mean of 634 residents. Between the least- and most-privileged quintiles, there was a pooled mean difference of $69,517 (CAD) in household income, 24 % in the fraction of adults without a high school diploma, 63 % in the fraction of visible minorities, 12 % in the fraction of recent immigrants, 17 % in the fraction of jobs not amenable to remote work, and 15 % in the fraction of suitable housing.

**Table 1:** Characteristics of the provincial populations.

Characteristic	Alberta Mean (min - max)	British Columbia Mean (min - max)	Ontario Mean (min - max)	Quebec^a^ Mean (min - max)
***Population sizes***				
DAs (N)	5802	7617	20,160	13,449
Total Population in 2016	4,067,175	4,648,055	13,448,494	7,437,202
Average DA Population	701 (<5–22,077)	610 (<5–8778)	671 (<5–16,747)	553 (<5–11,256)
***Frac. of adults without a high school diploma, quintiles (lowest to highest)***				
Least privileged [1]	0.26 (0.17–0.88)	0.23 (0.15–1.00)	0.24 (0.16–0.94)	0.29 (0.21–1.11)
Second-lowest [2]	0.14 (0.11–0.17)	0.12 (0.10–0.15)	0.13 (0.11–0.16)	0.17 (0.14–0.21)
Middle [3]	0.09 (0.07–0.11)	0.08 (0.07–0.10)	0.09 (0.07–0.11)	0.12 (0.09–0.14)
Second highest [4]	0.06 (0.04–0.07)	0.05 (0.04–0.06)	0.05 (0.04–0.07)	0.07 (0.05–0.09)
Most privileged [5]	0.02 (0.00–0.04)	0.01 (0.00–0.04)	0.01 (0.00–0.04)	0.02 (0.00–0.05)
***Avg. Income (CAD), quintiles (lowest to highest)***				
Least privileged [1]	36,996 (14,821–42,926)	40,534 (0–65,863)	24,798 (0–33,827)	25,401 (0–31,991)
Second-lowest [2]	47,212 (42,938–51,391)	74,191 (65,869–81646)	37,207 (33,828–40464)	34,562 (31,992–37,037)
Middle [3]	55,568 (51,398–60,242)	88,355 (81,647–95,186)	43,507 (40,465–46,829)	39,597 (37,038–42,383)
Second highest [4]	65,486 (60,244–72,978)	103,758 (95,232–113,993)	50,937 (46,833–56,142)	45,754 (42,385–49,999)
Most privileged [5]	108,293 (73,012–556,177)	150,974 (113,999–725,553)	79,931 (56,143-101,3494)	66,598 (50,001–846,434)
***Fraction of Visible Minority, quintiles (highest to lowest)***				
Least privileged [1]	0.57 (0.42–1.00)	0.77 (0.60–1.00)	0.77 (0.58–1.00)	0.43 (0.24–1.00)
Second-lowest [2]	0.32 (0.24–0.42)	0.44 (0.30–0.60)	0.41 (0.27–0.58)	0.15 (0.09–0.24)
Middle [3]	0.17 (0.11–0.24)	0.20 (0.12–0.30)	0.18 (0.11–0.27)	0.05 (0.03–0.09)
Second highest [4]	0.07 (0.04–0.11)	0.08 (0.04–0.12)	0.07 (0.03–0.11)	0.02 (0.00–0.03)
Most privileged [5]	0.01 (0.00–0.04)	0.02 (0.00–0.04)	0.01 (0.00–0.03)	0.00 (0.00–0.00)
***Fraction of Recent Immigrants, quintiles (highest to lowest)***				
Least privileged [1]	0.15 (0.09–0.50)	0.11 (0.07–0.41)	0.11 (0.06–0.41)	0.10 (0.04–0.48)
Second-lowest [2]	0.07 (0.05–0.09)	0.05 (0.04–0.07)	0.04 (0.03–0.06)	0.03 (0.02–0.04)
Middle [3]	0.03 (0.02–0.05)	0.03 (0.02–0.04)	0.02 (0.01–0.03)	0.01 (0.00–0.02)
Second highest [4]	0.02 (0.00–0.02)	0.01 (0.00–0.02)	0.01 (0.00–0.01)	0.00 (0.00–0.00)^b^
Most privileged [5]	0.00 (0.00–0.00)	0.00 (0.00–0.00)	0.00 (0.00–0.00)	0.00 (0.00–0.00)^b^
***Fraction of Essential Services Not Amenable To Remote Work, quintiles (highest to lowest)***				
Least-privileged [1]	0.99 (0.97–1.00)	0.99 (0.96–1.00)	0.99 (0.97–1.00)	0.99 (0.97–1.00)
Second-lowest [2]	0.96 (0.95–0.97)	0.95 (0.94–0.96)	0.96 (0.95–0.97)	0.96 (0.95–0.97)
Middle [3]	0.94 (0.93–0.95)	0.93 (0.96-0.91)	0.94 (0.93–0.95)	0.94 (0.93–0.95)
Second-highest [4]	0.91 (0.89–0.93)	0.90 (0.88–0.91)	0.91 (0.89–0.93)	0.92 (0.90–0.93)
Most-privileged [5]	0.79 (0.89-0.20)	0.81 (0.14–0.87)	0.84 (0.23–0.89)	0.85 (0.33–0.90)
***Fraction of Suitable Housing, quintiles (lowest to highest)***				
Least privileged [1]	0.86 (0.38–0.93)	0.85 (0.45–0.91)	0.83 (0.31–0.90)	0.88 (0.44–0.94)
Second-lowest [2]	0.94 (0.93–0.95)	0.93 (0.91–0.95)	0.93 (0.90–0.94)	0.95 (0.94–0.96)
Middle [3]	0.96 (0.95–0.97)	0.96 (0.95–0.97)	0.95 (0.94–0.97)	0.97 (0.96–0.99)
Second highest [4]	0.98 (0.97–0.99)	0.97 (0.97–0.99)	0.97 (0.97–0.99)	1.00 (1.00–1.00)^b^
Most privileged [5]	1.00 (1.00–1.00)	1.00 (1.00–1.00)	1.00 (1.00–1.00)	1.00 (1.00–1.00)^b^

a Population counts for Quebec include only persons aged 12 years and over.

b The upper 40 percent had identical scores.

### 3.1 Vaccination rate surges and differences between quintiles

**Fig. 1:**
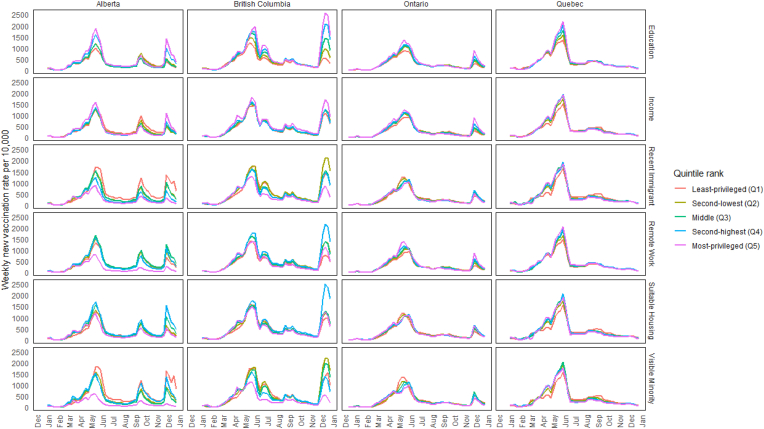
Incident rates of first-dose COVID vaccine administrations by social factor in 2021 across provinces Note: *Using Statistics Canada's Census data (2016*) *available at the time of database construction* [18]*, quintiles were generated using neighbourhoods (DA) within-province rank for (i) average household income (after-tax) and the fractions of (ii) adults with high school diplomas, (iii) recent immigrants, (iv) visible minorities, (v) suitable housing, and (vi) those with occupations amenable to remote working.*

**Fig. 2:**
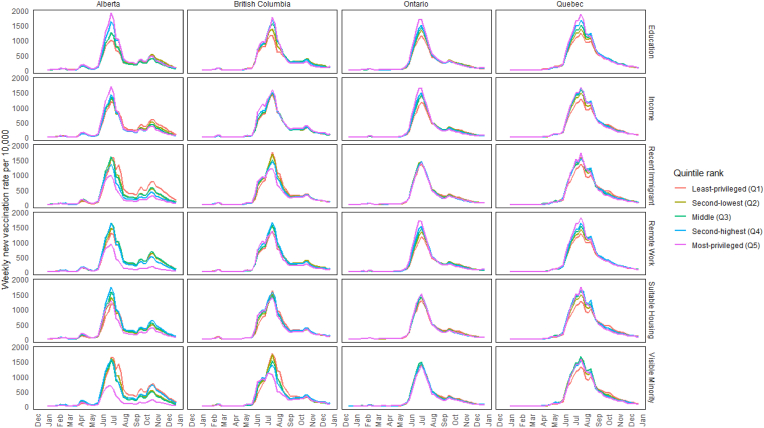
Incident rates of second-dose COVID vaccine administrations by social factor in 2021 across provinces Note: *Using Statistics Canada's Census data (2016) available at the time of database construction* [18]*, quintiles were generated using neighbourhoods (DA) within-province rank for (i) average household income (after-tax) and the fractions of (ii) adults with high school diplomas, (iii) recent immigrants, (iv) visible minorities, (v) suitable housing, and (vi) those with occupations amenable to remote working.*

There were peaks in absolute differences between quintiles across all four provinces during three major surges in vaccination rates in all provinces, typically reflected about 2 weeks after a change in vaccination eligibility. The first surge followed the opening of first-dose vaccinations for the 12–17 age group in late May^1^ 2021 (Fig. 1). The weekly vaccination rate for the least- and most-privileged quintiles respectively when ranked by education was 1002 versus 1625 first doses per 10,000 unvaccinated residents during Alberta's peak on May 22; 974 versus 1972 on June 5 in British Columbia; 892 versus 1345 on May 22 in Ontario; and 1363 versus 2179 on June 5 in Quebec. The second surge followed the opening of second-dose vaccinations for this and other age groups^2^ (Fig. 2). The rate for second doses for the least-versus most-privileged quintiles when ranked by education was 1010 versus 1907 per 10,000 on July 3 in Alberta, 1173 versus 1758 on July 24 in British Columbia, 1159 versus 1708 on July 10 in Ontario, and 1236 versus 1872 on July 24 in Quebec. The third surge followed the opening of first-dose vaccinations for the 5–11 age group following Health Canada approval on November 19. Vaccination rates for the least- and most-privileged quintiles when ranked by education was 381 versus 1402 per 10,000 on December 4 in Alberta, 544 versus 2562 in British Columbia on December 18, and 309 versus 876 on December 4 in Ontario (Fig. 1; Quebec data excluded this age group).

When ranking quintiles by all other factors, the magnitude of differences between quintiles varied across western and eastern provinces (Fig. 1, Fig. 2). Most notable are the prominent surges in the western provinces, especially Alberta, following an announcement of mandatory vaccination policy for all employees at healthcare facilities, a $100 vaccination incentive on September 3, and the enforcement of proof of vaccination for accessing non-essential services beginning on September 15 [4]. When ranking by all variables except education, Alberta's highest quintile was the least impacted, whereas the spread between quintiles was more compressed in the eastern provinces. For example, the peak first-dose rate during this fall surge was reached on September 25 in Alberta's least-versus most-privileged quintiles when ranked by the fraction of recent immigrants was 1224 versus 420 per 10,000, respectively, but was 243 versus 223 in that same week (Fig. 1, Fig. 2).

### 3.2 Differences between quintiles in cumulative vaccination coverage

**Fig. 3:**
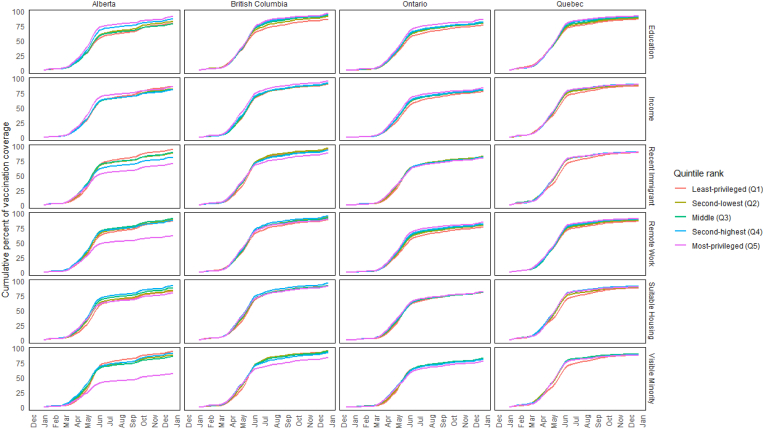
Diffusion curves of the first-dose of COVID vaccines by social factor in 2021 across provinces Note: *Using Statistics Canada's Census data (2016) available at the time of database construction* [18], *quintiles were generated using neighbourhoods (DA) within-province rank for (i) average household income (after-tax) and the fractions of (ii) adults with high school diplomas, (iii) recent immigrants, (iv) visible minorities, (v) suitable housing, and (vi) those with occupations amenable to remote working.*

**Fig. 4:**
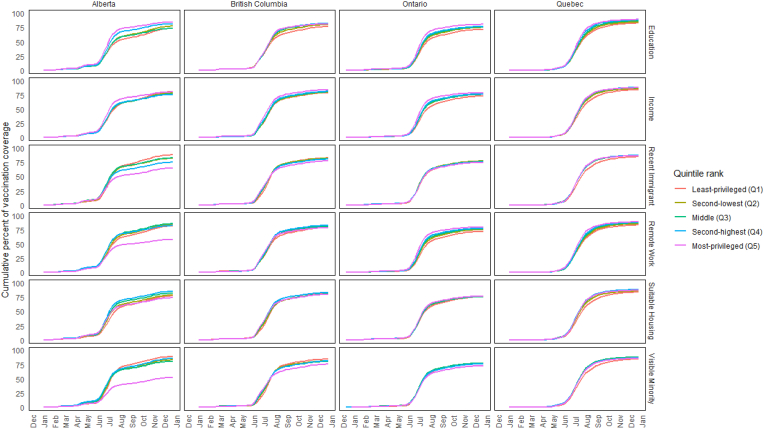
Diffusion curves of the second-dose of COVID vaccines by social factor in 2021 across provinces Note: *Using Statistics Canada's Census data (2016) available at the time of database construction* [18], *quintiles were generated using neighbourhoods (DA) within-province rank for (i) average household income (after-tax) and the fractions of (ii) adults with high school diplomas, (iii) recent immigrants, (iv) visible minorities, (v) suitable housing, and (vi) those with occupations amenable to remote working.*

Differences in vaccination coverage that were driven by these surges became clearly pronounced in July for the first dose and August for the second dose (Fig. 3, Fig. 4). For example, first-dose vaccination coverage on July 17, 2021, comparing the least-to the most-privileged areas by education quintiles, was 61 % vs 77 % in Alberta, 72 % vs 85 % in British Columbia, 63 % vs 75 % in Ontario, and 75 % vs 84 % in Quebec. Second-dose vaccination coverage on August 14, 2021 in the least-versus most-privileged education quintiles was 54 % vs 73 % in Alberta, 59 % vs 70 % in British Columbia, 57 % vs 70 % in Ontario and 62 % vs 75 % in Quebec. The unique pronounced differences between Alberta's most-advantaged quintile versus all others were also apparent when ranked by the fraction of visible minorities, recent immigrants, and remote work. For example, vaccination coverage in the lowest versus highest quintiles ranked by the fraction of visible minorities was 45 % vs 80 %, respectively, for the first dose on July 17 and 40 % vs 71 %, respectively, for the second dose on August 14.

### 3.3 Gini coefficient across the quintiles over time

**Fig. 5:**
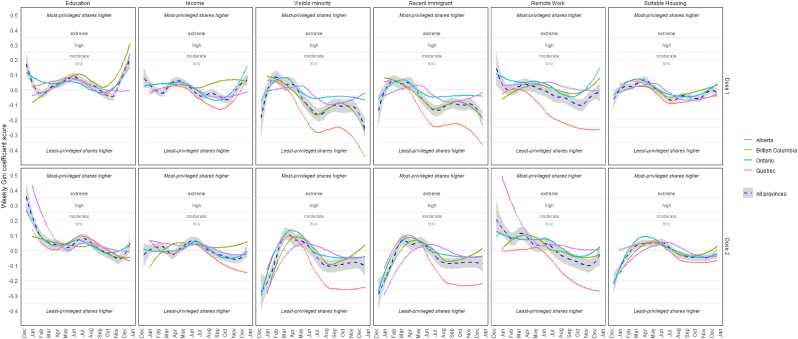
Weekly Gini coefficient scores across and provinces by marker of social or economic advantage in 2021 Note: *Gini coefficients were based on the number of new vaccinations* per *10,000 persons* per *quintile. A perfectly proportionate distribution has a Gini coefficient of 0. Values closer to 1 suggest vaccines were disproportionately allocated to higher ranking populations. Similar to a Concentration Index measure* [20], *values closer to -1 suggest vaccines were disproportionately allocated to lower ranking populations. Gini coefficients below 0.20 are considered a low degree of inequality, 0.25-0.35 is moderate, 0.35-0.50 is high, and over 0.50 is extreme* [19]*. The blue dashed line represents the average among the four provinces.*

The Gini coefficient fluctuated throughout the year and differed across provinces and area-level measures of privilege (Fig. 5). The disproportionalities generally oscillated within relatively low ranges, either favoring the more privileged quintiles (i.e., Gini Index scores between 0.00 and 0.20), such as those ranking higher in education, or the less privileged quintiles (i.e., Gini Index scores between 0.00 and −0.20), such as those ranking higher in the fractions of recent immigrants or visible minorities. However, a consistency across all provinces was that there were disproportionately more first-dose administrations in the most-privileged quintiles in the week of April 3, but this pattern was reversed by the end of May when quintiles were ranked by the fractions of recent immigrants and visible minorities (with the exception of Quebec); this same phenomenon was also observed for second-dose vaccinations for the week of May 1 (with some exceptions in Alberta and Ontario). In three provinces (Alberta, Ontario, Quebec), there were disproportionately more first- and second-dose vaccinations in less-privileged quintiles during five consecutive weeks, stretching from the weeks ending on October 9 through November 6, regardless of the ranking variable.

### 3.4 Vaccination coverage by social indicators

Alberta showed unique patterns when compared to other provinces, particularly Ontario and Quebec, across all determinants. When ranked by education, Alberta displayed the largest gaps in cumulative vaccination coverage, with first-dose coverage on July 17 ranging from 61 % in the least-privileged quintile to 77 % in the most-privileged quintile, compared to smaller gaps in other provinces (e.g., 72 %–85 % in British Columbia, 63 %–75 % in Ontario, and 75 %–84 % in Quebec). Rankings by household income revealed first-dose coverage gaps as large as 20 percentage points in Alberta on July 17 (63 % in the lowest quintile vs. 83 % in the highest quintile), while other provinces showed smaller but still notable gaps (e.g., 70 %–82 % in British Columbia). Rankings by the fraction of visible minorities revealed disparities across provinces, with Alberta showing the widest gaps (e.g., 45 %–80 % on July 17 for first-dose coverage), while other provinces exhibited smaller differences of 9–15 percentage points. For recent immigrants, Alberta's September 25 surge showed a stark contrast in first-dose rates, with neighbourhoods in the lowest quintile achieving 1224 vaccinations per 10,000 unvaccinated residents compared to 420 per 10,000 in the highest quintile. Rankings by jobs amenable to remote work and suitable housing revealed less pronounced differences, with gaps under 10 percentage points across provinces still highlighting measurable heterogeneity.

### 3.5 Sensitivity analysis - vaccination coverage in urban alberta

**Fig. 6:**
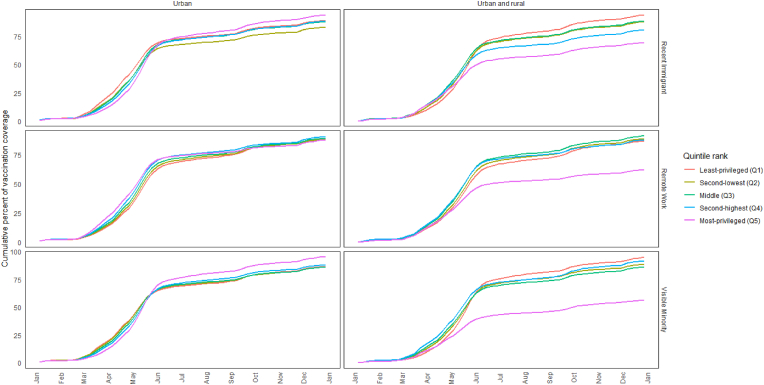
Diffusion curves of the first-dose COVID vaccines in Alberta only by social factor in all regions versus urban only (excluding rural areas) Note: *A sensitivity analysis was performed to evaluate whether the extraordinarily low vaccination coverage observed in the main analysis (right column) among Alberta's most-advantaged neighbourhoods was driven by rural regions. Diffusion curves for neighbourhoods in Calgary and Edmonton exclusively are plotted in the left column, which confirmed the suspicion.*

Sensitivity analysis confirmed that when the quintiles were ranked for Calgary and Edmonton, vaccination coverage was highest among the most-privileged urban areas when ranking by the fractions of recent immigrants or visible minorities, similarly to other provinces (Fig. 6); however, this difference was not as pronounced. On December 25, vaccination coverage when ranking quintiles by these two factors (i.e., the fractions of recent immigrants and visible minorities) in the most-privileged urban areas reached 94 % and 95 % as compared to 89 % and 86 % least-privileged ones; these same indicators were separated by 24 percentage points (69 % versus 93 %) and 38 percentage points (94 % versus 56 %), respectively, when analysis included urban and rural areas analyzed together. When ranking by the fraction of jobs amenable to remote work, the least- and most-privileged urban areas were at 89 % versus 88 %, respectively, only a percentage point difference; this same percent difference was 25 (87 % versus 62 %) when urban and rural areas were analyzed together.

## 4 Discussion

Our study observed that when neighbourhoods were ranked into quintiles by the fraction of high school graduates, there were relatively low (i.e., Gini coefficients below 0.20), but persistent disproportionalities in the number of COVID-19 vaccination administrations in 2021 across the four provinces, including during key moments when vaccination rates were highest in May–June (following the opening of vaccinations for all 12+ age groups) and November–December (following the opening of vaccinations for the 5–11 age group). The cumulative effect of small disparities between quintiles (i.e, moderate Gini coefficients) was apparent by mid-summer of 2021 with cumulative coverage differences 9–16 percent lower in the bottom quintile as compared to the top quintile. When ranking by other measures, however, the results varied between eastern and western provinces. A uniquely large surge of vaccinations appeared to occur in Alberta, but not elsewhere. Alberta's neighbourhoods that ranked as most advantaged by the fraction of visible minorities, recent immigrants, or by remote work had the lowest vaccination coverage observed by our study, which appears to have been driven by rural neighbourhoods.

Disparities in vaccine uptake were discernible when quintiles were ranked by educational attainment across all provinces and both doses. Educational attainment is frequently discussed as one of the most influential and powerful social determinants of health [3]. Our results align with other studies from the United States finding that education was the strongest area-level predictor of vaccination and booster uptake after controlling for age, as compared to average income and the percentage of racialized minorities or essential workers [14]. This finding is also consistent with studies of other COVID-19 interventions such as testing [21,22]. This phenomenon may be the result of disparities in the number of pharmacies providing vaccination services in neighbourhoods with lower levels of educational attainment or other markers of social disadvantage [9]. Another contributing factor may be that the work opportunities that come with higher education may generally come with a greater job flexibility to seek out vaccination opportunities, and secure and attend scheduled appointments; jobs requiring higher levels of education may also be associated with urban areas with easier access to vaccination sites [23].

While other inferential studies have controlled for age and still found education to be a powerful predictor [14], a reasonable alternative explanation in our results is that lower neighbourhood-levels of educational attainment could correlate to areas where younger adults and families reside. However, recent Statistics Canada individual-level surveys conducted have confirmed the association with lower education after controlling for age [24]. Furthermore, our study observed that some of the most pronounced social differences between quintiles followed the opening of vaccinations for the 5–11 age group in November 2021. A notable concern here is that inequities in vaccine coverage observed in adults was mirrored, or even magnified, in their children. The same advantages associated with the adult populations may be compounded for children, i.e., parents with flexible jobs and/or childcare.

Our study observed some notable differences between provinces. For example, Alberta saw a uniquely large surge in vaccinations in September 2021, especially in the most disadvantaged quintiles. While Alberta introduced workplace mandates and vaccine passport policies similar to other provinces, it uniquely implemented a controversial $100 incentive program in early September. Although some have questioned its impact based on preliminary data [25], rigorous analyses have not yet been available. It is possible that this incentive may have played a role in contributing to this surge, particularly when combined with other measures [26]. This incentive was distinct from measures in Ontario and Quebec, where such a noticeable rise in vaccination rates was not observed [16]. Future studies should evaluate the extent to which Alberta's incentive program contributed to its September surge and consider its broader implications for vaccine strategies, particularly in disadvantaged areas [27,28].

A stark difference was also observed in Alberta's vaccine coverage between most- and least-advantaged quintiles when ranked by the fraction of visible minorities or recent immigrants (i.e., the opposite pattern compared to ranking by education). While targeted efforts, such as “hot spot” strategies to vaccinate areas considered to be disproportionately at risk for COVID-19 (i.e., Calgary's northeast quadrant), may explain some of these trends [7], our sensitivity analysis revealed rurality was a major driver. Vaccine hesitancy, known to be more prevalent in Alberta's rural and religious communities, likely contributed to this contrast [29]. This hesitancy has also been observed in British Columbia, but Alberta's distinctive socio-political climate, which includes strong libertarian values and distrust of government interventions, may amplify this phenomenon [30,31]. Addressing hesitancy in these areas could involve working with community leaders [32], providing alternative vaccine options (e.g., non-MRNA-based vaccines), or offering non-vaccine-based treatments, such as nirmatrelvir/ritonavir (Paxlovid). However, the persistent under-utilization of therapies like Paxlovid in Alberta calls into question the potential of relying on such alternatives [33]. These findings suggest that rural regions in Alberta may require tailored, trust-building approaches, as well as contingency planning to manage the heightened risks of severe or long COVID-19 outcomes during periods of high transmission.

The value of trust-building and localized approaches for vaccine acceptance has been widely recognized internationally, with evidence supporting the translatability of these principles across national income strata [34]. For instance, studies in South Africa and Nigeria have demonstrated lower vaccine uptake in socioeconomically disadvantaged regions, often driven by trust deficits and limited healthcare access in those same regions [12,34]. Similarly, research in Ethiopia emphasizes the value of culturally tailored, community-based interventions to address structural and social barriers to vaccination [13]. While some principles may translate, addressing vaccination disparities in non-high-income countries—or vice versa—still requires careful consideration of the unique cultural, structural, logistical, and social challenges inherent to each context [13,34,35].

While this investigation had many strengths (e.g., it was a cross-provincial collaboration representing more than 84 % of the Canadian population), it was also subject to several limitations. First, our study is situated within the context of high-income provinces in Canada, characterized by universal healthcare access and robust vaccine supply chains, as noted above. This can limit generalizability to the Global South. Further, our study could not provide age- and sex-stratified results, given the constraints introduced by sharing data across provincial borders. Without age- and sex-stratified data, our findings do not capture differences in vaccine uptake across demographic subgroups, such as the lower responsiveness of younger males observed in other studies [36]; therefore, caution is needed when using this study to inform population groupings not defined by neighbourhoods. Similarly, while our sensitivity analysis was stratified by rurality, resources and data sharing agreements did not allow such a stratification for all participating provinces, which may further limit generalizability. Second, the most recent census data available at the time of study was the 2016 edition. If available resources and data sharing agreements allowed for an update with the 2021 census, neighbourhoods falling at the extreme quintile ranks may switch categorizations to be a rank above or below what was recorded in our study. However, such reclassifications are unlikely to significantly change our overall conclusions because quintile differences are driven by the most common traits of the neighbourhood groups within each quintile, not the individual ones falling near the dividing lines between ranks. Finally, our study did not track whether the observed heterogeneity across areas in their risk for severe or long-COVID-19 outcomes translated to disparities in health outcomes. We hope to address each of these limitations in future research.

## 5 Conclusion

Area-level disparities in vaccination coverage between neighbourhoods with high versus low fractions of high school graduates were observed across all provinces within six months of the COVID-19 vaccine availability, leaving some groups at greater risk of severe and longer-lasting health impacts. To better safeguard these communities, vaccine program planning could consider working in partnership neighborhoods at higher risk to tailor vaccination prioritization strategies. This may be particularly important during periods of surging provincial vaccination rates or campaigns targeting younger populations, such as when a vaccine is newly available. However, uptake in some rural western areas is likely to remain limited unless measures are taken to work collaboratively with these communities to reduce hesitancy, explore alternative vaccine options or approaches acceptable to these populations, and proactively address risks during periods of elevated transmission in those areas.

## Contributions to knowledge

What does this study add to existing knowledge?

● We consistently observed area-level COVID-19 vaccine coverage disparities when ranking neighbourhoods by the proportion of high school graduates in Canada's four largest provinces (Alberta, British Columbia, Ontario, and Quebec). These differences were largest when vaccination rates were highest following vaccination rollout for all 12+ age groups in May–June and for the 5–11 age group in November–December 2021.
● Yet when neighbourhoods were ranked by other metrics (the fractions of visible minorities, recent immigrants, or jobs amenable to remote work), results differed between eastern and western provinces, especially in Alberta where the most advantaged neighbourhoods had the lowest vaccination coverage.

What are the key implications for public health interventions, practice or policy?

● Canadian neighbourhoods with low-versus high-levels of education corresponded to low-versus high-levels of vaccine-induced protection from severe COVID-19, which may have reinforced health inequities in 2021.
● Devoting additional vaccination outreach services to neighbourhoods ranking lowest by education levels may be advisable, especially when vaccination rates are high and immediately following eligibility changes for minors.
● Localized strategies and health system planning that acknowledges the socio-political and cultural diversity influencing vaccine acceptance may be necessary nationwide, particularly in western rural regions.

## Consent to participate

Not applicable.

## Ethics approvals

Ethics approvals were obtained from the University of British Columbia Research Ethics Board (H20-02097), the University of Calgary Health Research Ethics Board (REB20-0688, REB19-1369_MOD1) and the Health Information Privacy Committee of Alberta Health (No. 2020/2021-32), the University of Toronto Health Sciences Research Ethics Board (39253) and the Institutional Review Board of McGill University in Quebec (A06-M52-20B).

## Ethics approvals

Ethics approvals were obtained from the University of British Columbia Research Ethics Board (H20-02097), the University of Calgary Health Research Ethics Board (REB20-0688, REB19-1369_MOD1) and the Health Information Privacy Committee of Alberta Health (No. 2020/2021-32), the University of Toronto Health Sciences Research Ethics Board (39253) and the Institutional Review Board of McGill University in Quebec (A06-M52-20B).

## Consent for publication

Not applicable.

## Availability of data and material

Not applicable.

## Code availability

Not applicable.

## Author contributions

Conceptualization: TW, SDB, NZJ, AK, MM-G, SM, RFB Data curation: AD’S, JLFA, HM, HA, FY, Formal analysis: NZJ, AK, JL, HA, MM-G, ERM, KM, SM, GM, HAVG, FY, RFB Funding acquisition: TW, SDB, JC, EC, NZJ, AK, MM-G, SM, RFB Methodology: TW, SDF, NZJ, AK, MM-G, SM, FY, RFB Project administration: RFB Supervision: RFB, TW Visualization: RFB Writing - original draft: RFB Writing - review & editing: *All authors*.

## Funding

This study was funded by the 10.13039/100021597Centre for Research on Pandemic Preparedness and Health Emergencies at the Canadian Institutes of Health Research (Funding Reference Number: PEE-183994)

## Declaration of competing interest

The authors declare the following financial interests/personal relationships which may be considered as potential competing interests: MM-G reports past contractual agreements with the I*nstitut national d'excellence en santé et services sociaux* (INESSS) and the *Institut national de santé publique du Québec* (INSPQ).

## Footnotes

1 Vaccinations for the 12–17 age group opened on May 10 in Alberta, May 20 in British Columbia; May 23, and May 25 in Quebec. See Canadian Institutes for Health Information. COVID-19 Intervention Scan: Canadian Institutes for Health Information; 2022 [Available from: https://www.cihi.ca/en/covid-19-intervention-scan].

2 Those who received first-doses in May became second-dose eligible starting on June 28; the province sent second-dose invitations 6–7 weeks following first-dose in British Columbia. See CBC News. Getting your 2nd shot: What British Columbians need to know 2021 [Available from: https://www.cbc.ca/news/canada/british-columbia/second-dose-bc-vaccine-what-you-need-to-know-1.6052002]; those who received a first-dose vaccination prior to May 31 became eligible for a second dose on June 23; and those age 12 and older became second-dose eligible on July 9. See Canadian Institutes for Health Information. COVID-19 Intervention Scan: Canadian Institutes for Health Information; 2022 [Available from: https://www.cihi.ca/en/covid-19-intervention-scan].
